# Diverse Daily Activity Patterns: Examining Daily Activity Sequences and Activity Diversity in Older Adults

**DOI:** 10.1093/geroni/igaf122.1603

**Published:** 2025-12-31

**Authors:** Rachel Koffer, Sandra Duezel, Denis Gerstorf, Ilja Demuth, Ulman Lindenberger, Soomi Lee, Johanna Drewelies

**Affiliations:** Arizona State University, Phoenix, Arizona, United States; Charité - Universitätsmedizin Berlin, Berlin, Berlin, Germany; Humboldt University Berlin, Humboldt University Berlin, Berlin, Germany; Charité - Universitätsmedizin Berlin, Berlin, Berlin, Germany; Max Planck Institute for Human Development, Berlin, Berlin, Germany; The Pennsylvania State University, University Park, Pennsylvania, United States; Humboldt University Berlin, Humboldt University Berlin, Berlin, Germany

## Abstract

While daily activity diversity has been widely associated with healthy aging outcomes, further examination of daily activity patterns can uncover daily time use that is most beneficial for older adults. This study identified distinct daily activity sequences and examined whether they were associated with better quality sleep that night. Using a sample of older adults who participated in an experience sampling study embedded into the Berlin Aging Study II (N = 140, Mage = 77.09, range: 67–88 years), we applied sequence analysis to self-reported activity data (social, mental, leisure, physical, self-care, domestic, and productive activities) collected six times per day over seven consecutive days (N = 879 daily sequences). Distance metrics identified how dissimilar each day’s sequence was from each other day. Using cluster analysis of the distance metrics, we identified 8 common sequence patterns. Days with activity patterns dominated by physical and domestic work predicted better quality sleep than days with patterns dominated by leisure (B = -2.10, p = .004), self-care (B = -5.33, p < .001), social (B = -3.65, p < .001), and mental activities (B = -2.91, p = .01). Findings controlled for comorbidities, age, gender, education, and weekday. Further, the likelihood of each sequence pattern differed across age and traditional measures of activity diversity. Daily activities in later life vary not just in diversity of types but also in the sequential patterns in which they occur throughout the day. Findings highlight the benefit of examining daily activity sequences in addition to activity diversity.

